# IMPACT_S: Integrated Multiprogram Platform to Analyze and Combine Tests of Selection

**DOI:** 10.1371/journal.pone.0096243

**Published:** 2014-10-20

**Authors:** Emanuel Maldonado, Kartik Sunagar, Daniela Almeida, Vitor Vasconcelos, Agostinho Antunes

**Affiliations:** 1 CIIMAR/CIMAR – Interdisciplinary Centre of Marine and Environmental Research, University of Porto, Porto, Portugal; 2 Department of Biology, Faculty of Sciences, University of Porto, Porto, Portugal; University of California, San Diego, United States of America

## Abstract

Among the major goals of research in evolutionary biology are the identification of genes targeted by natural selection and understanding how various regimes of evolution affect the fitness of an organism. In particular, adaptive evolution enables organisms to adapt to changing ecological factors such as diet, temperature, habitat, predatory pressures and prey abundance. An integrative approach is crucial for the identification of non-synonymous mutations that introduce radical changes in protein biochemistry and thus in turn influence the structure and function of proteins. Performing such analyses manually is often a time-consuming process, due to the large number of statistical files generated from multiple approaches, especially when assessing numerous taxa and/or large datasets. We present IMPACT_S, an easy-to-use Graphical User Interface (GUI) software, which rapidly and effectively integrates, filters and combines results from three widely used programs for assessing the influence of selection: Codeml (PAML package), Datamonkey and TreeSAAP. It enables the identification and tabulation of sites detected by these programs as evolving under the influence of positive, neutral and/or negative selection in protein-coding genes. IMPACT_S further facilitates the automatic mapping of these sites onto the three-dimensional structures of proteins. Other useful tools incorporated in IMPACT_S include Jmol, Archaeopteryx, Gnuplot, PhyML, a built-in Swiss-Model interface and a PDB downloader. The relevance and functionality of IMPACT_S is shown through a case study on the toxicoferan-reptilian Cysteine-rich Secretory Proteins (CRiSPs). IMPACT_S is a platform-independent software released under GPLv3 license, freely available online from http://impact-s.sourceforge.net.

## Introduction

The nature and strength of evolutionary selection pressures can be estimated at the molecular level, as a non-synonymous to synonymous substitution rate ratio omega (*ω = dN/dS*), where *ω* greater than, equal to and less than 1 is indicative of positive, neutral and negative selection, respectively [1]. This approach often fails to detect subtle adaptations that only affect certain regions of the protein and/or take place over a very short period of evolutionary time [2]. Moreover, the evaluation of selective pressures solely at the nucleotide level and the assumption that all mutations affect the fitness of the organism equally could be misleading. Although non-synonymous substitutions introduce variations in coding regions, a novel amino acid could have identical or similar biochemical and/or structural properties to that of the ancestral residue. Such substitutions are unlikely to influence the structure or the function of the protein and hence are least likely to affect the fitness of an organism. Thus, it is important to discern the nature of mutations to precisely understand the evolution of a protein. By employing mapping strategies of mutational sites onto the three-dimensional (3D) structure of the protein, it is possible to gain further insights into how its structure and/or function are affected by the changes in certain residues or regions [3]. For example, using a similar integrative approach, we and others have demonstrated that most predatory venom-components in a diversity of animal lineages adopt Rapid Accumulation of Variations in Exposed Residues of Toxins [4] and accumulate mutations on the molecular surface under the influence of positive Darwinian selection, while preserving the key functional and structural residues that stabilize the overall structure of the protein [4]–[13]. The mutation of the molecular surface may increase the toxin’s ability to target novel molecular receptors and aid in evading immune response upon injection into prey animals [8]. Phenomena like this can be easily detected by mapping sites under positive and negative selection on 3D structures of proteins. Mapping of sites onto multiple sequence alignments of protein-coding genes is also beneficial, as it enables the identification of differential evolution of domains by revealing mutational hotspots [5], [10], [11]. Hence, to efficiently assess the influence of natural selection on protein-coding genes and to accurately identify regions under various regimes of natural selection, it is essential to adopt a complementary approach [14]. Although several software applications have been proposed to independently evaluate selection pressures at the codon-level [15]–[18], an integrative approach, which additionally evaluates the strength and radicalness of non-synonymous substitutions at the amino acid-level, is still missing. In addition, the manual integration of results from various approaches is time-consuming.

To address these shortcomings and to facilitate the integration of results from various selection assessments, we propose IMPACT_S, a free platform-independent user-friendly GUI software that integrates results of nucleotide and amino acid-level assessments by employing three widely used softwares: Codeml from Phylogenetic Analysis by Maximum Likelihood (PAML) package [17], Datamonkey, a web-server of the HyPhy [20] package - www.datamonkey.org [18], [19], and Selection on Amino Acid Properties using phylogenetic trees (TreeSAAP) [15]. Sites detected as positively and negatively selected can be automatically mapped onto the 3D structure of the protein. If experimentally determined protein structures are unavailable, IMPACT_S facilitates the prediction of models, through homology, using the built-in Swiss-Model [21]–[23] interface. Homology modeling is based on evolutionary relationships between target and template sequences. The template sequences result from homology searches of experimentally determined protein structures, through successive BLAST-p [34] searches. This method involves the construction of an atomic resolution model for the protein under study using an experimentally determined 3D-structure of a similar homologous protein [23]. The following criteria are automatically considered by Swiss-Model in order to obtain a single high quality template: (i) identification of related proteins with experimentally solved structures, (ii) mapping of corresponding residues of target and template structures, (iii) building of the 3D model on the basis of the alignment and finally, (iv) evaluation of the quality of the resulting model [23].

In the following sections, we introduce the foundations, the structure and the development of IMPACT_S and demonstrate the relevance and functionality of IMPACT_S using a case study on Cysteine-rich Secretory Proteins (CRiSPs), a class of reptilian and mammalian glycoproteins with extremely diversified functions. CRiSPs have been theorized to play important functions in the mammalian reproductive pathways, while in toxicoferan reptiles (venomous snakes and lizards) they have been hypothesized to participate in prey envenoming and capture [11].

## Design and Implementation

### IMPACT_S Foundation

IMPACT_S integrates three broadly used bioinformatic softwares for assessing selection pressures shaping the evolution of protein-coding genes: (i) PAML and (ii) Datamonkey for assessing selection at the codon-level, and (iii) TreeSAAP to detect selection at the protein-level.

Codeml from the PAML package [17], implements powerful site-specific [24], [25], branch-specific [26], [27] and branch-site specific models [25], [28] that detect the influence of natural selection, easily and reliably. Site-specific models, which are the current focus of this work, comprise the alternative models – model 2a (M2a) and model 8 (M8) and the null models – model 1a (M1a) and model 7 (M7). The alternative models include the Bayes Empirical Bayes (BEB) [25] approach for identifying positively selected sites. The computed *ω* value and the sites detected by the alternative models as positively selected are only considered by the user if the likelihood ratio test (LRT) is significant [24], [25]. The LRT is conducted by comparing the null models with the alternative models (M1a *vs.* M2a and/or M7 *vs.* M8). Both M2a and M8 have one additional class or category when compared to their null counterparts M1a and M7, respectively [24]. In case the LRT is significant in both tests, with the alternative models as fitting the data better, then it is possible to assume stronger evidence for the presence of sites under positive selection [25]. However, PAML is incapable of identifying negatively selected sites, in contrast to certain models implemented in the Datamonkey web-server [18], [19]: Single-Likelihood Ancestor Counting (SLAC) [16], Fixed Effects Likelihood (FEL) [16], Random Effects Likelihood (REL) [16] and Fast Unconstrained Bayesian AppRoximation (FUBAR) [29] methods. The recently proposed Mixed Effects Model of Evolution (MEME) [2] is a state-of-the-art method for detecting sites that evolve under episodic selection pressures, which are often difficult to identify using traditional site-specific methods. This method allows the distribution of *ω* to vary not only across sites, but also from branch to branch at a site [2]. SLAC infers the number of non-synonymous and synonymous substitutions that have occurred at each site using Maximum Likelihood (ML) reconstructions of ancestral sequences, while FEL estimates the ratio of non-synonymous to synonymous substitutions, not assuming *a priori* distribution of rates across sites substitution, on a site-by-site basis. REL involves fitting a distribution of substitution rates across sites and then inferring the rate at which individual sites evolve based on ML estimates. FUBAR on the other hand, which supersedes SLAC, FEL and REL, infers the rate at which individual sites evolve based on an approximate hierarchical Bayesian approach using a Markov Chain Monte Carlo sampler.

The choice of the method not only depends on the question being addressed, but also on the size of the dataset. When the positive selection on the evolution of protein-coding genes is sufficiently strong, the site-specific models of Codeml [17], [25], [30] can efficiently identify positively selected sites. However, due to the episodic or transient nature of natural selection on protein-coding genes, the precise identification of the regions that have undergone adaptive evolution is often difficult [2]. Moreover, a strong influence of purifying selection on a majority of lineages can mask the signal of positive selection on others [2]. MEME was proposed to address these shortcomings and to reliably identify sites that are influenced by both episodic and pervasive influence of positive selection at the level of an individual site [2]. In order to identify sites influenced by positive and negative selection, the following methods can be chosen, considering the size of datasets to avoid biased results [16]: SLAC for large datasets (over 40 sequences), REL for datasets of intermediate size (20–40 sequences), FEL for intermediate to large datasets (over 50 sequences) and FUBAR for analyzing very large datasets (the authors tested this model on a dataset of 3142 sequences, which is much faster when compared with the other methods [29]). While Datamonkey and Codeml assess the influence of natural selection at the codon-level, TreeSAAP measures the selective influences on 31 biochemical and structural amino acid properties – such as hydrophobicity, polarity, solvent accessible reduction ratio and buriedness – during cladogenesis and performs goodness-of-fit and categorical statistical tests [15]. It enables the estimation of radicalness of a mutation at a particular site by revealing the influence of the novel amino acid, introduced by non-synonymous nucleotide substitution, on the structural and biochemical properties of the protein. The result files generated by TreeSAAP are organized in two main subdirectories: Evpthwy and Substs. The former includes the sliding window analyses, while the latter contains results for particular sites [31], [32]. Both identify amino acid properties under selection, scored in every category, typically from 1 to 8. The assessment of biochemical and structural properties of the novel amino acids, introduced by non-synonymous substitutions, results in generation of numerous statistical files, especially when a large number of amino acid properties are evaluated. In order to detect sites that recurrently fall under the influence of natural selection or to detect sites with a defined number of selected properties, the user has to assess an array of statistical files. Thus, the interpretation of the result files generated by TreeSAAP for downstream analyses can be an extremely time-consuming process.

Manually noting down log-likelihood (*lnL*) values, various maximum-likelihood parameter estimates and *ω* values from the result files of Codeml for every model analyzed is also tedious. These values are essential for conducting likelihood-ratio tests and the identification of the appropriate regime of selection (for additional information regarding models and LRT tests, see [24]). This process is required to be repeated for each dataset being analyzed. Similarly, tables generated by the Datamonkey web-server, which contain various site statistics, are required to be manually downloaded and processed to retrieve sites detected as positively and negatively selected at the desired significance level.

It has been suggested that the application of all the major selection assessment methods in HyPhy [20] (SLAC, FEL, REL, FUBAR and MEME), followed by the consensual identification of sites detected as positively selected among the methods, can minimize false positives, especially when analyzing smaller datasets [16]. This feature is indeed available on the Datamonkey web-server. However, the results of these analyses can only be integrated manually with the results of Codeml and TreeSAAP selection assessments and the compilation of the sites detected in common by these approaches becomes a difficult task. Moreover, to our knowledge, there is no software to export these results for the downstream analyses, such as the mapping of the detected mutational sites onto the 3D structures of proteins.

Therefore, IMPACT_S has been designed to be a single platform, with which the user can employ various state-of-the-art selection assessments and identify the regime of natural selection on protein-coding genes. Furthermore, these sites could be automatically mapped onto the alignment and 3D structure (either in a homology model or a known crystallographic structure of the protein), making the entire process extremely rapid and efficient, even when a large number of datasets are analyzed.

### IMPACT_S Structure

IMPACT_S implements four main tabs: (i) ‘PAML’, (ii) ‘Datamonkey’, (iii) ‘TreeSAAP’ and (iv) ‘Results & 3D’. The first three tabs enable the user to import the corresponding result files and automatically extract the sites detected as positively and negatively selected. IMPACT_S has been designed to organize these results in Comma Separated Values (CSV) format, which can be viewed and exported with the built-in CSV Viewer tool. Sites detected as positively and negatively selected by various selection assessment methods can be mapped individually for each method, or mapped in combination, as common sites, onto the 3D structure of proteins under the ‘Results and 3D’ tab. To map sites from two or more methods (considering the type of selection) in conjunction, as common sites, these must be firstly merged into a final Merged Results (MR) table. IMPACT_S provides several mapping schemes (please refer to Table 4 in the tutorial included in the package, for a list of all the available schemes and descriptions), which are applied to the results and presented using the molecular visualization software, Jmol [33] (http://jmol.sourceforge.net/). The 3D structure can be obtained through homology modeling using the built-in Protein Modeler tool that acts as an interface to the Swiss-Model web-server [21]–[23] (http://swissmodel.expasy.org). Alternatively, these sites can be mapped onto the experimentally deduced crystal structures by importing PDB files from RCSB Protein Data Bank (RCSB PDB) [35], [36] (http://www.pdb.org) or the Swiss-Model Template Library (SMTL or ExPDB) [21]–[23] into IMPACT_S. Sites can also be marked onto the two-dimensional (2D) codon or protein sequence alignment using the built-in Alignment Filter tool. This useful feature enables the assessment of the influence of domain-specific accumulation of mutations and helps to generate publication quality figures (Figure 10 in [3]). This tool is available in every tab of IMPACT_S, except in the TreeSAAP Evpthwy, where the user can find the built-in Gnuplot (http://www.gnuplot.info/) Options tool, which is specifically designed to plot graphs for the sliding window analysis using any amino acid property file. IMPACT_S also incorporates phylogenetic resources such as PhyML [37] and Archaeopteryx (http://www.phylosoft.org/archaeopteryx), the successor of ATV [38], and other built-in tools that deal with Multiple Sequence Alignment (MSA) and CSV files [39]. Throughout the process of dataset analyses the user may have different requirements and hence employ these tools to perform any alignment and/or phylogenetic tree alterations, as well as the generation of new phylogenetic trees, which are required for PAML and TreeSAAP and optional in Datamonkey analyses.

### IMPACT_S Development

The IMPACT_S program was implemented in Java and has been tested on Linux, Windows and MacOS workstations. Java SwingWorker threads (from Java version 1.6.0) are used to run the entire integrated software, handle outputs and to allow GUI updates. Even though it is possible to process and filter PAML results using BioPerl libraries, it is a difficult task for users with no programming skills. Several tools found in IMPACT_S were improved from our previously developed software IMPACT [39], which include the MSA Editor/Viewer, the MSA Format Converter and the CSV Viewer. IMPACT_S uses the Internet to connect to the Swiss-Model [21]–[23] and RCSB PDB [35], [36] (http://www.pdb.org/) web-servers. The functionalities related to these web-servers are found in two distinct built-in tools, the Protein Modeler tool which implements Swiss Model functionalities and the PDB Downloader tool which includes the RSCB PDB and the SMTL.

### IMPACT_S Availability and Requirements

IMPACT_S software was developed in Java with the platform-independent context in mind, providing the use of the software by a wider audience of users. The source code can be used by anyone with skills to manipulate and extend the program capabilities, or even used in other projects. It is an open source software distributed under GNU General Public License version 3.0 (GPLv3) and is freely available with an example dataset, a detailed tutorial and manual, to allow its user-friendly application, at http://impact-s.sourceforge.net. IMPACT_S is an integrated platform, enabling users to access results and knowledge from any stage of the user controlled workflow [40]. Being an integrated platform, users are not required to install independent programs, since they are already provided in the download package, but are allowed to replace (or include) the existing binaries. IMPACT_S requires the following:

Operating System: Linux/UNIX, Windows or MacOS;

Programming Language: Java;

Optional Programs Included: Codeml (PAML), TreeSAAP, PhyML, Archaeopteryx, Jmol and Gnuplot;

Other Requirements: Java version (minimum) 1.6.0.

## Results

CRiSPs belong to a class of glycoproteins exclusive to vertebrates and have been implicated in a broad range of functions. Previously, we have demonstrated that toxicoferan-reptilian CRiSPs are significantly influenced by positive selection, and in snakes more than Anguimorpha and helodermatid lizards, while their mammalian homologues exhibit extreme coding sequence conservation (Table S1) [11]. By using a toxicoferan-reptilian CRiSPs dataset we demonstrate the applications of IMPACT_S and its useful designs and features. It should be noted that we have used the same dataset only to present all the tabs and associated methods. Nonetheless, in a real evolutionary study the researcher must choose the method that best fits the dataset.

**Figure 1:**
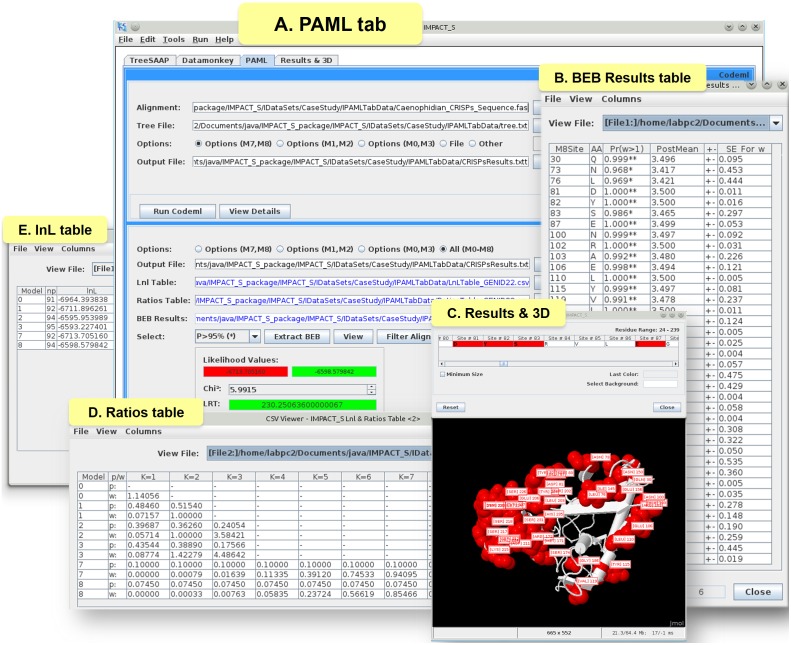
PAML tab and related results. (A) The **‘**PAML’ tab from IMPACT_S showing the results of the LRT test (M7 *vs.* M8), (B) Automatically extracted and tabulated ‘BEB results’ table, (C) Mapping of the positively selected sites onto the 3D protein-structure of CRiSPs (2GIZ-A), (D) Tabulated results of *ω* estimation under the site-specific models (M0, M1, M3, M7 and M8) and (E) The ‘lnL’ table, showing all the log-likelihood values for these models.

Site-specific analyses were executed under the ‘PAML’ tab (Figure 1A) of IMPACT_S, and the value of *ω* was estimated using the following pairs of models: i) M0 and M3; ii) M1a and M2a and iii) M7 and M8. IMPACT_S was then used to automatically extract all the relevant information from the generated result files, such as a table of *ω,* the ML parameter estimates associated with each model (Figure 1D) and *lnL* table for every model containing the corresponding number of parameters (*np*) (Figure 1E). The LRT was conducted in IMPACT_S to compare alternate models that allow *ω*>1 (M3, M2a and M8) with their null models that do not (M0, M1a and M7). The user can select from three significance thresholds, namely: *p* = 0.05 (by default selected), *p* = 0.01 and *p* = 0.001. The degrees of freedom (*df*) are calculated and may differ according to the pair of models selected due to the different *np*. For example, if the pairs (M1a *vs.* M2a) or (M7 *vs.* M8) are selected the *df* is 2. The (M0 *vs.* M3) pair is an exception, for which the *df* is 4. The sites detected as evolving under positive selection according to the BEB [25] approach (Figure 1B) were then extracted using IMPACT_S and automatically mapped onto the 3D structure of CRiSPs (PDB: 2GIZ-A; Figure 1C).

**Figure 2:**
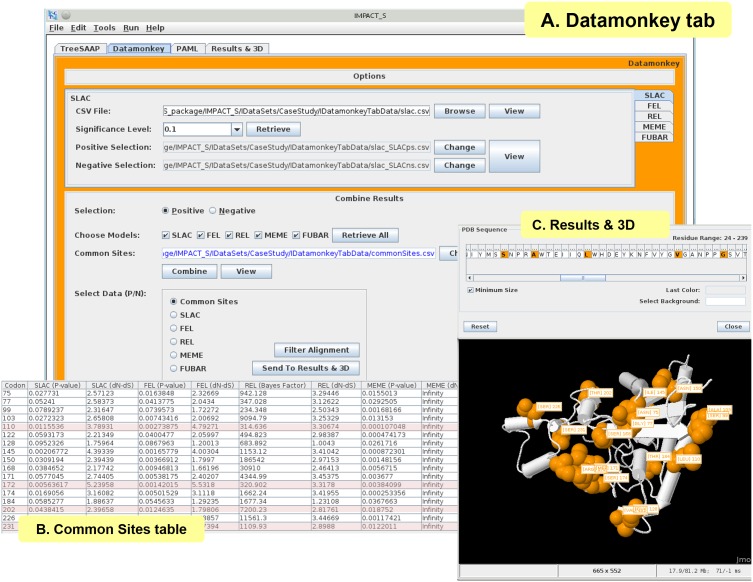
Datamonkey tab and related results. (A) ‘Datamonkey’ tab showing all the selected options, (B) ‘Common Sites’ table generated from the positive-selection analyses under various methods (SLAC, FEL, REL, MEME and FUBAR) – The sites 110, 172, 202 and 231 (highlighted in pink), were simultaneously detected by Datamonkey, PAML (Codeml) and TreeSAAP as positively-selected (Table S1) [1], (C) ‘Results & 3D’ tab showing the mapping of sites found in the ‘Common Sites’ table.

All the models implemented in the Datamonkey web-server (SLAC, FEL, REL, MEME and FUBAR) were executed and the corresponding results were processed under the ‘Datamonkey’ tab of IMPACT_S (Figure 2A). Using the default significance cut-off, the results of positive and negative selection analyses were tabulated automatically for each method. The default significance cut-offs used in IMPACT_S are the same as on the Datamonkey web-server: *p* = 0.1 for SLAC, FEL and MEME; posterior probability = 0.9 for FUBAR and Bayes Factor = 50 for REL. The significance given by default can be changed by selecting from the available options or by typing the desired value. The positively selected sites detected using the default significance under these methods were tabulated, combined (Figure 2B: ‘Common Sites’ table) and then mapped onto the 3D structure of the protein (Figure 2C) in IMPACT_S.

**Figure 3:**
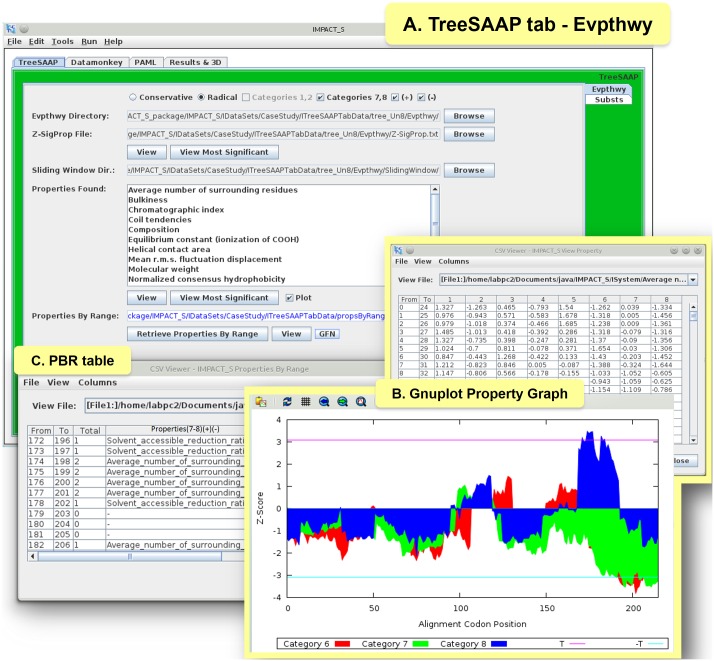
Evolutionary Pathway (Evpthwy) tab under TreeSAAP tab and related results. (A) ‘TreeSAAP’ tab showing the ‘Evpthwy’ tab with results of the CRiSP case study, (B) Gnuplot graph for the property “Average number of surrounding residues” with Z-scores in y-axis and Codon positions in x-axis, (C) ‘PBR’ table showing all ranges (‘From’ - ‘To’) retrieved from the sliding window analysis with the ‘Total’ count of properties for each range and their names [Properties (7–8) (+) (−) – names of properties under significant positive (Z-score ≥3.09) and negative (Z-score ≤3.09) selection, found in the categories 7 and 8].

**Figure 4:**
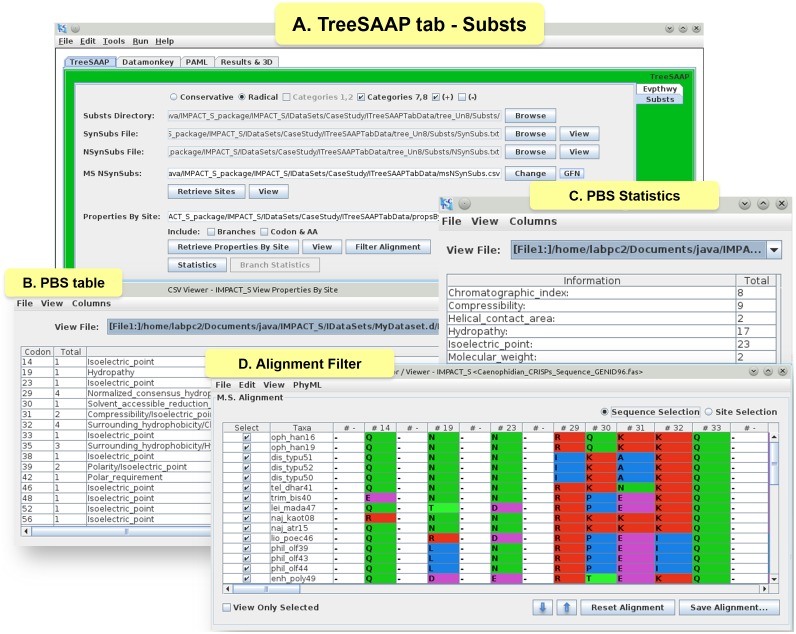
Substitutions (Substs) tab under TreeSAAP tab and related results. (A) ‘TreeSAAP’ tab showing the ‘Substs’ tab and the results of the CRiSPs case study, (B) ‘PBS’ table showing all the significant codons (‘Codon’ column) and the total count (‘Total’ column) of properties for each codon and their respective names [‘Properties (7–8) (+)’ – names of properties under significant (Z-score ≥3.09) positive selection], (C) ‘PBS statistics’, with respect to the PBS table, showing the number of times (‘Total’ column) that the same property (‘Information’ column) is selected across the data set provided and (D) Alignment Filter option with the final view of the alignment containing only the codons from the PBS table with their original positions.

Furthermore, we conducted a protein-level analyses under the ‘TreeSAAP’ tab to measure the influence of selection on 31 biochemical properties. Here, the data was processed from the ‘Evpthwy’ tab for the sliding window analyses and from the ‘Substs’ tab for mapping the substitutions on the 3D structure of the protein. In the Evpthwy directory under ‘TreeSAAP’ tab (Figure 3A), the ‘Properties By Range (PBR)’ table was generated (Figure 3B), which consists of the most significant properties under categories 7 and 8 (most radical changes). A graph was then plotted using the Gnuplot Options dedicated tool for each significant property (Figure 3C). The ‘Substs’ tab (Figure 4A) was used to generate the ‘Properties By Site (PBS)’ table (Figure 4B) that consists of all the significant codons under categories 7 and 8. This tab provides the statistical files (Figure 4C) for counting properties. Using the ‘Alignment Filter’ tool, the sites reported in the PBS table as positively selected were highlighted in a new alignment view that keeps only these sites and their original positions in the provided alignment (Figure 4D). The information regarding the radical physicochemical amino acid changes varying across the reptilian-CRiSPs phylogenetic tree can also be obtained under this tab (Table S2).

**Figure 5:**
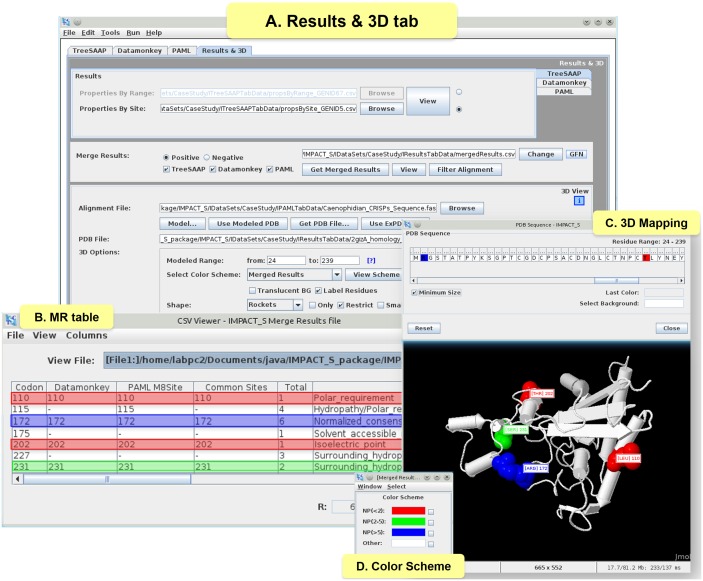
3D mapping and related functionalities under Results & 3D tab. (A) **‘**Results & 3D’ tab with the results of the CRiSPs case study, (B) ‘MR table’ (Table S3) presenting six columns: ‘Codon’ containing the site found as positive selected (TreeSAAP – Z-score ≥3.09); ‘Datamonkey’ with the positive selected common sites positions from SLAC, FEL, REL, MEME and FUBAR; ‘PAML M8Site’ with the information from BEB results M8; ‘Common Sites’ – reporting the site position common to all the previous mentioned columns; ‘Total’ - count of the number of properties per site; ‘Properties (7–8) (+)’ with the names of properties found under categories 7 and 8 for Z-score ≥3.09, (only common sites are shown and highlighted) (C) 3D structure and ‘PDB Sequence’, mapping the sites positions from the ‘Common Sites’ column following the (D) Merged Results ‘Color Scheme’ according to a pre-defined number of properties: red – less than 2 (NP<2), green - between 2 and 5 (NP(2–5)) and blue - more than 5 (NP>5).

In order to identify sites that were detected in common as positively selected by the aforementioned analyses (PAML, Datamonkey and TreeSAAP), their resultant files were combined in IMPACT_S under the ‘Results & 3D’ tab (Figure 5A) as the MR table (Figure 5B; Table S3). The evolution of such sites are most likely to be influenced by natural selection, since they are detected in common by a diversity of selection assessment methodologies. Such sites are then automatically mapped onto the 3D structure (2GIZ-A; Figure 5C) using the ‘Merged Results’ Color Scheme option (Figure 5D).

## Conclusion

A diversity of selection assessment methodologies are employed to identify and corroborate sites that are influenced by various regimes of natural selection. Analyzing and integrating the result files from these methods is a tedious and time-consuming process. In this context, IMPACT_S offers a user-friendly interface to rapidly manage, filter, organize and interpret the results in different ways. Moreover, IMPACT_S provides the user the freedom to choose different available methods, which should follow the respective authors’ recommendations from programs integrated in this GUI. If properly used, this software will automatically map the positively and negatively selected sites onto the sequence alignment as well as the 3D protein structure. This enables the user to infer which sites are important, according to their specific location and significance for all types of selection. IMPACT_S is a new integrated multiprogram platform, which allows the user to combine the results from different bioinformatic programs. Merging PAML or Datamonkey with TreeSAAP may provide additional information about amino acid properties, such as hydrophobicity, at the sites identified by the aforementioned codon methods. Overall, amino acid properties can provide crucial information that can be extremely relevant in protein-protein interactions [41] and thus a change in this biochemical property can compromise the folding of the protein interfering in its stability and function.

## Supporting Information

Table S1 Amino-acid sites under positive selection in toxicofera-reptilian CRiSPs.
(DOCX)

Table S2 IMPACT_S count of the positively-selected properties varying across the toxicofera-reptilian CRiSPs phylogenetic tree.
(DOCX)

Table S3 Merged results from the IMPACT_S integrative approach for the analyses of toxicofera-reptilian CRiSPs.
(DOCX)
