# Supplementary material for: IMPACT_S: Integrated Multiprogram Platform to Analyze and Combine Tests of Selection
Source: PLoS One. 2014 Oct 20;9(10):e96243. doi: 10.1371/journal.pone.0096243 (PMC4203653; doi:10.1371/journal.pone.0096243)
Supplement: Table S1 — Amino-acid sites under positive selection in toxicofera-reptilian CRiSPs. (DOCX) [file pone.0096243.s001.docx]

**Table S1.** Amino-acid sites under positive selection in toxicofera-reptilian CRiSPs.

| **Sites** | | **PAML** | | **TreeSAAP**  **Radical changes in amino-acid properties** | | | |
| --- | --- | --- | --- | --- | --- | --- | --- |
| **Position** | **A.A** | **M2a** | **M8** | **Chemical** | **Structural** | **Total** | **A.S.A** |
| **Snakes** | |  |  |  |  |  |  |
| 30 | Q | 3.495 ± 0.12 | 3.496 ± 0.095 | - | α_C_ V^0^ | 2 | **92.9%** |
| 73 | N | 3.392 ± 0.512 | 3.417 ± 0.453 | - | α_C_ | 1 | **57.6%** |
| 75 | N | 3.31 ± 0.664 | - | - | α_C_ | 1 | 35.0% |
| 76 | L | 3.396 ± 0.444 | 3.421 ±0.444 | - | α_C_ | 1 | 7.0% |
| 81 | D | 3.501 ±0.011 | 3.5 ±0.011 | - | α_C_ | 1 | **75.9%** |
| 82 | Y | 3.501 ±0.016 | 3.5 ±0.016 | - | α_C_ B_I_ | 2 | **78.9%** |
| 83 | S | 3.454 ±0.297 | 3.465 ±0.297 | - | α_C_ B_I_ | 2 | 37.5% |
| 87 | E | 3.499 ±0.053 | 3.499 ±0.053 | - | α_C_ B_I_ | 2 | **57.7%** |
| 100 | N | 3.496 ±0.092 | 3.497 ±0.092 | pK’ | α_C_ | 2 | **82.6%** |
| 102 | R | 3.5 ± 0.031 | 3.5 ± 0.031 | pK’ | α_C_ | 2 | 28.2% |
| 103 | A | 3.472 ±0.226 | 3.48 ± 0.226 | pK’ | α_C_ | 2 | **64.3%** |
| 106 | E | 3.492 ±0.121 | 3.494 ±0.121 | pK’ | α_C_ | 2 | **47.2%** |
| 110 | L | 3.501 ±0.005 | 3.5 ±0.005 | pK’ | α_C_ R_a_ N_S_ H_P_ | 5 | 32.9% |
| **115** | **Y** | **3.497 ±0.081** | **3.497 ±0.081** | **pK’** | **α_C_ R_a_ P_β_ N_S_ H_P_** | **6** | **47.1%** |
| **119** | **V** | **3.471 ±0.237** | **3.478 ±0.237** | **pK’** | **α_C_ R_a_ P_β_ N_S_ H_P_** | **6** | **53.2%** |
| 145 | I | 3.501 ±0.011 | 3.5 ±0.011 | pK’ | α_C_ R_a_ P_β_ H_P_ | 5 | 0% |
| 150 | N | 3.492 ±0.124 | 3.494 ±0.124 | pK’ | α_C_ R_a_ P_β_ H_P_ | 5 | 22.9% |
| 156 | E | 3.501 ±0.005 | 3.5 ±0.005 | pK’ | α_C_ R_a_ P_β_ H_P_ | 5 | **63.9%** |
| 168 | S | 3.282 ± 0.706 | - | pK’ | αC Ra Pβ Hnc NS HP | 7 | 21.7% |
| **171** | **M** | **3.5 ±0.025** | **3.5 ±0.025** | **pK’** | **Α_C_ R_F_ R_a_ P_β_ H_nc_ N_S_ H_P_** | **8** | **45.9%** |
| **172** | **R** | **3.501 ±0.004** | **3.5 ±0.004** | **-** | **α_C_ B_I_ R_F_ R_a_ P_β_ H_nc_ N_S_ H_P_** | **8** | **87.0%** |
| **174** | **S** | **3.499 ±0.057** | **3.499 ±0.057** | **-** | **α_C_ B_I_ R_F_ R_a_ P_β_ H_nc_ N_S_ H_P_** | **8** | **54.4%** |
| **186** | **G** | **3.386 ±0.475** | **3.409 ±0.475** | **-** | **α_C_ B_I_ P_C_ R_F_ R_a_ P_β_ H_nc_ N_S_ H_P_** | **9** | **41.7%** |
| **202** | **T** | **3.402 ±0.429** | **3.426 ±0.429** | **-** | **α_C_ B_I_ P_C_ R_F_ R_a_ P_β_ N_S_ α_m_ H_P_** | **9** | **97.9%** |
| **203** | **L** | **3.501 ±0.004** | **3.5 ±0.004** | **-** | **α_C_ B_I_ P_C_ R_F_ R_a_ P_β_ N_S_ α_m_ H_P_** | **9** | **65.1%** |
| **204** | **Y** | **3.499 ±0.058** | **3.499 ±0.058** | **-** | **α_C_ B_I_ P_C_ R_F_ R_a_ P_β_ α_m_** | **7** | 33.6% |
| 206 | E | 3.501 ±0.004 | 3.5 ±0.004 | - | α_C_ B_I_ R_a_ P_β_ α_m_ | 5 | **40.0%** |
| 207 | Y | 3.45 ±0.308 | 3.462 ±0.308 | - | α_C_ P_β_ α_m_ | 3 | 35.3% |
| 211 | D | 3.445 ±0.322 | 3.459 ± 0.322 | - | α_C_ α_m_ | 2 | **88.7%** |
| 212 | S | 3.499 ±0.05 | 3.499 ±0.05 | - | α_C_ α_m_ | 2 | **55.9%** |
| 214 | V | 3.356 ±0.535 | 3.383 ±0.535 | - | α_C_ α_m_ | 2 | **47.6%** |
| 215 | K | 3.432 ±0.36 | 3.448 ±0.36 | - | α_C_ α_m_ | 2 | **78.1%** |
| 217 | S | 3.501 ±0.005 | 3.5 ±0.005 | - | α_C_ α_m_ | 2 | **45.0%** |
| 218 | S | 3.5 ±0.035 | 3.5 ±0.035 | - | α_C_ | 1 | **60.0%** |
| 220 | Q | 3.46 ±0.278 | 3.469 ±0.278 | - | α_C_ | 1 | **95.6%** |
| 222 | E | 3.254 ± 0.746 | - | - | α_C_ | 1 | **90.8%** |
| 223 | W | 3.488 ±0.148 | 3.491 ±0.148 | - | α_C_ | 1 | **55.7%** |
| 224 | I | 3.481 ±0.19 | 3.486 ±0.19 | - | α_C_ | 1 | 4.1% |
| 226 | S | 3.465 ±0.259 | 3.473 ±0.259 | - | α_C_ | 1 | **60.2%** |
| 231 | S | 3.394 ±0.445 | 3.42 ±0.445 | - | α_C_ | 1 | 19.9% |
| 235 | H | 3.501 ±0.019 | 3.5 ±0.019 | - | α_C_ | 1 | **84.3%** |

**Note:** Amino-acid sites detected by PAML and TreeSAAP under positive selection along with the ω estimation and Bayesian (BEB) analysis posterior probabilities for sites with P ≥ 95% under M2a and M8 models. Sites simultaneously identified as positively-selected by nucleotide and protein-level assessment are highlighted in bold. **TreeSAAP**: Radical changes in amino-acid properties (chemical, structural and other property changes) under category 6 and/or 7 and/or 8. **Amino-acid property symbols used:** Average number of surrounding residues ***(Ns)***, β-structure tendencies ***(Pβ)***, Bulkiness ***(Bl)***, Composition ***(c),*** Chromatographic index ***(RF)***, Coil tendencies ***(Pc)***, Equilibrium constant for ionization of COOH ***(pK’)***, Isoelectric point ***(pHi)***, Normalized consensus hydrophobicity ***(Hnc)***, Partial specific volume ***(V^0^)***, Polar requirement ***(Pr )*,** Power to be at C-terminus of the *α*-helix **(*αc*)**, Power to be in the middle of an *α*-helix ***(αm),*** Solvent accessible reduction ratio ***(Ra)***, Surrounding hydrophobicity **(*Hp*).**
