# Supplementary material for: IMPACT_S: Integrated Multiprogram Platform to Analyze and Combine Tests of Selection
Source: PLoS One. 2014 Oct 20;9(10):e96243. doi: 10.1371/journal.pone.0096243 (PMC4203653; doi:10.1371/journal.pone.0096243)
Supplement: Table S2 — IMPACT_S count of the positively-selected properties varying across the toxicofera-reptilian CRiSPs phylogenetic tree. (DOCX) [file pone.0096243.s002.docx]

**Table S2.** IMPACT_S count of the positively-selected properties varying across the toxicofera-reptilian CRiSPs phylogenetic tree.

| **A) Node to Branch** | **Unique Properties** | **All Properties** |
| --- | --- | --- |
| node#71_-->_trim_fla26: | 2 | 3 |
| node#72_-->_agk_blom27: | 4 | 4 |
| node#64_-->_lei_mada47: | 6 | 10 |
| node#74_-->_sis_cate36: | 6 | 9 |
| node#82_-->_hop_step09: | 1 | 1 |
| node#57_-->_lio_poec46: | 5 | 8 |
| node#70_-->_Trim_ste14: | 3 | 3 |
| node#60_-->_trim_bis40: | 3 | 4 |
| node#78_-->_oph_han32: | 3 | 3 |
| node#54_-->_cer_ryn04: | 1 | 1 |
| node#74_-->_crot_atr20: | 4 | 4 |
| node#67_-->_naj_kaot08: | 1 | 1 |
| node#86_-->_rhin_nig33: | 1 | 1 |
| node#71_-->_trim_jer18: | 1 | 2 |
| node#68_-->_vip_buru30: | 3 | 4 |
| node#84_-->_trop_car34: | 1 | 1 |
| node#53_-->_cer_ryn02: | 1 | 1 |
| node#73_-->_agk_pis21: | 5 | 7 |
| node#80_-->_dem_vest35: | 1 | 1 |
| **B) Node to Node** | **Unique Properties** | **All Properties** |
| node#47_-->_node#76: | 10 | 19 |
| node#68_-->_node#69: | 6 | 7 |
| node#80_-->_node#81: | 3 | 3 |
| node#83_-->_node#84: | 1 | 1 |
| node#65_-->_node#67: | 3 | 3 |
| node#47_-->_node#75: | 6 | 9 |
| node#64_-->_node#65: | 2 | 3 |
| node#49_-->_node#50: | 1 | 1 |
| node#70_-->_node#71: | 3 | 3 |
| node#60_-->_node#61: | 1 | 1 |
| node#52_-->_node#53: | 1 | 1 |
| node#78_-->_node#79: | 3 | 3 |
| node#69_-->_node#70: | 1 | 1 |
| node#88_-->_node#89: | 1 | 1 |
| node#48_-->_node#49: | 1 | 1 |
| node#51_-->_node#57: | 4 | 6 |
| node#57_-->_node#58: | 5 | 6 |
| node#76_-->_node#80: | 1 | 1 |
| node#69_-->_node#72: | 1 | 1 |
| node#85_-->_node#88: | 6 | 7 |
| node#86_-->_node#87: | 3 | 3 |
| node#61_-->_node#62: | 7 | 14 |
| node#81_-->_node#82: | 1 | 1 |
| node#49_-->_node#64: | 1 | 1 |
| node#47_-->_node#48: | 3 | 3 |
| node#62_-->_node#63: | 2 | 2 |
| node#48_-->_node#68: | 5 | 5 |
| node#51_-->_node#52: | 6 | 7 |
| node#52_-->_node#56: | 3 | 3 |
| node#72_-->_node#73: | 5 | 5 |

**Note:** Positively-selected radical physicochemical amino acid changes varying across the toxicofera-reptilian CRiSPs phylogenetic tree. ‘Node-Species’ table (A) and ‘Node-Node’ table (B), showing the unique and all properties count in each case - both associated with the ‘PBS’ table resultant from TreeSAAP tab – Substs.
