# Supplementary material for: IMPACT_S: Integrated Multiprogram Platform to Analyze and Combine Tests of Selection
Source: PLoS One. 2014 Oct 20;9(10):e96243. doi: 10.1371/journal.pone.0096243 (PMC4203653; doi:10.1371/journal.pone.0096243)
Supplement: Table S3 — Merged results from the IMPACT_S integrative approach for the analyses of toxicofera-reptilian CRiSPs. (DOCX) [file pone.0096243.s003.docx]

**Table S3.** Merged results from the IMPACT_S integrative approach for the analyses of toxicofera-reptilian CRiSPs.

| Codon | Datamonkey | PAML M8Site | Common Sites | Total | Properties(7-8)(+) |
| --- | --- | --- | --- | --- | --- |
| 14 | - | - | - | 1 | Isoelectric_point |
| 19 | - | - | - | 1 | Hydropathy |
| 23 | - | - | - | 1 | Isoelectric_point |
| 29 | - | - | - | 4 | Normalized_consensus_hydrophobicity/Chromatographic_index/Hydropathy/Solvent_accessible_reduction_ratio |
| 30 | - | 30 | - | 1 | Solvent_accessible_reduction_ratio |
| 31 | - | - | - | 2 | Compressibility/Isoelectric_point |
| 32 | - | - | - | 4 | Surrounding_hydrophobicity/Chromatographic_index/Hydropathy/Solvent_accessible_reduction_ratio |
| 33 | - | - | - | 1 | Isoelectric_point |
| 35 | - | - | - | 3 | Surrounding_hydrophobicity/Hydropathy/Isoelectric_point |
| 38 | - | - | - | 1 | Isoelectric_point |
| 39 | - | - | - | 2 | Polarity/Isoelectric_point |
| 42 | - | - | - | 1 | Polar_requirement |
| 46 | - | - | - | 1 | Isoelectric_point |
| 48 | - | - | - | 1 | Isoelectric_point |
| 52 | - | - | - | 1 | Isoelectric_point |
| 56 | - | - | - | 1 | Isoelectric_point |
| 58 | - | - | - | 1 | Isoelectric_point |
| 60 | - | - | - | 1 | Polarity |
| 62 | - | - | - | 1 | Isoelectric_point |
| 65 | - | - | - | 1 | Polar_requirement |
| 68 | - | - | - | 1 | Isoelectric_point |
| 72 | - | - | - | 2 | Polar_requirement/Polarity |
| 73 | - | 73 | - | 1 | Hydropathy |
| 75 | 75 | - | - | 2 | Solvent_accessible_reduction_ratio/Polarity |
| 76 | - | 76 | - | 2 | Polar_requirement/Polarity |
| 81 | - | 81 | - | 2 | Normalized_consensus_hydrophobicity/Isoelectric_point |
| 82 | - | 82 | - | 1 | Polarity |
| 87 | - | 87 | - | 1 | Compressibility |
| 90 | - | - | - | 1 | Isoelectric_point |
| 97 | - | - | - | 1 | Polarity |
| 100 | - | 100 | - | 3 | Surrounding_hydrophobicity/Hydropathy/Solvent_accessible_reduction_ratio |
| 101 | - | - | - | 1 | Solvent_accessible_reduction_ratio |
| 102 | - | 102 | - | 4 | Normalized_consensus_hydrophobicity/Chromatographic_index/Hydropathy/Solvent_accessible_reduction_ratio |
| 105 | - | - | - | 1 | Solvent_accessible_reduction_ratio |
| 106 | - | 106 | - | 4 | Molecular_weight/Helical_contact_area/Compressibility/Partial_specific_volume |
| **110** | **110** | **110** | **110*** | **1** | **Polar_requirement** |
| 115 | - | 115 | - | 4 | Hydropathy/Polar_requirement/Solvent_accessible_reduction_ratio/Polarity |
| 119 | - | 119 | - | 2 | Polar_requirement/Polarity |
| 125 | - | - | - | 3 | Surrounding_hydrophobicity/Hydropathy/Solvent_accessible_reduction_ratio |
| 144 | - | - | - | 4 | Normalized_consensus_hydrophobicity/Chromatographic_index/Hydropathy/Polarity |
| 151 | - | - | - | 1 | Polarity |
| 156 | - | 156 | - | 4 | Chromatographic_index/Hydropathy/Compressibility/Isoelectric_point |
| 168 | 168 | - | - | 1 | Solvent_accessible_reduction_ratio |
| **172** | **172** | **172** | **172*** | **6** | **Normalized_consensus_hydrophobicity/Surrounding_hydrophobicity/Chromatographic_index/Hydropathy/Solvent_accessible_reduction_ratio/Compressibility** |
| 175 | - | - | - | 1 | Solvent_accessible_reduction_ratio |
| 191 | - | - | - | 1 | Polar_requirement |
| 193 | - | - | - | 5 | Surrounding_hydrophobicity/Hydropathy/Polar_requirement/Solvent_accessible_reduction_ratio/Polarity |
| 195 | - | - | - | 3 | Molecular_weight/Helical_contact_area/Partial_specific_volume |
| **202** | **202** | **202** | **202*** | **1** | **Isoelectric_point** |
| 203 | - | 203 | - | 4 | Surrounding_hydrophobicity/Hydropathy/Solvent_accessible_reduction_ratio/Isoelectric_point |
| 204 | - | 204 | - | 1 | Isoelectric_point |
| 205 | - | - | - | 4 | Surrounding_hydrophobicity/Chromatographic_index/Hydropathy/Solvent_accessible_reduction_ratio |
| 206 | - | 206 | - | 4 | Surrounding_hydrophobicity/Hydropathy/Solvent_accessible_reduction_ratio/Isoelectric_point |
| 210 | - | - | - | 1 | Chromatographic_index |
| 211 | - | 211 | - | 1 | Compressibility |
| 212 | - | 212 | - | 1 | Isoelectric_point |
| 215 | - | 215 | - | 2 | Compressibility/Isoelectric_point |
| 222 | - | - | - | 1 | Compressibility |
| 224 | - | 224 | - | 2 | Polar_requirement/Polarity |
| 227 | - | - | - | 3 | Surrounding_hydrophobicity/Hydropathy/Solvent_accessible_reduction_ratio |
| **231** | **231** | **231** | **231*** | **2** | **Surrounding_hydrophobicity/Solvent_accessible_reduction_ratio** |
| 235 | - | 235 | - | 1 | Compressibility |
| 239 | - | - | - | 1 | Polarity |

**Note:** ‘Merged Results’ table from ‘Results & 3D’ tab, showing the four sites positively-selected by the IMPACT_S integrative approach (TreeSAAP - green, Datamonkey - organge and PAML - blue) highlighted in bold and containing an (*) in the ‘Common Sites’ column.
